# Microbial trash to metabolic treasure

**DOI:** 10.1097/IN9.0000000000000067

**Published:** 2025-07-21

**Authors:** Alexander S. Dowdell, Sean P. Colgan

**Affiliations:** 1 Mucosal Inflammation Program and Division of Gastroenterology and Hepatology, Department of Medicine, University of Colorado, Aurora, CO, USA;; 2 Department of Medicine, Rocky Mountain Veterans Regional VA Medical Center, Aurora, CO, USA

**Keywords:** macrophage, phagocytosis, mTORC1, AMPK, autophagy, neutrophil, dendritic cell

## Abstract

In a recent *Nature* publication, Lesbats et al uncover the molecular fate of phagocytosed bacterial contents. The authors observed incorporation of bacterial biomolecules (amino acids, metabolites) into those of the host macrophage through stable isotope labeling and mass spectrometry. Further, the authors found that the state of the phagocytosed bacteria, living or dead, dramatically alters the macrophage’s metabolic program toward either a pro-inflammatory or a “recycling” direction, respectively. This commentary summarizes these findings and further discusses the implications of this work in a broader sense.

Macrophages (MΦ) are a heterogeneous population of immune cells with essential and diverse biological roles, including tissue/organ maintenance, pathogen surveillance, and immune regulation ^[1–3]^. Foremost among their characteristic functions is the engulfment and isolation of external matter (such as microbes) through a process termed “phagocytosis”. Such material is enclosed in specialized organelles called “phagosomes”, which fuse with the lysosome for degradation of the phagocytosed matter ^[4]^.

While phagocytosis was first observed as far back the mid-1800s, it is remarkable to imagine that the fate of engulfed cargo remains incompletely understood. In recent work, Lesbats et al demonstrate that biomolecules from phagocytosed and degraded avirulent *Escherichia coli* are incorporated into the host MΦ ^[5]^. Utilizing metabolic labeling of bacterial amino acids and metabolites through stable carbon isotope ^13^C, then tracing the fate of labeled biomolecules by liquid chromatography (LC) - or ultra high performance liquid chromatography (UHPLC)-coupled mass spectrometry (M/S), the authors found that phagocytosis of killed *E. coli* (KEC) resulted in efficient labeling of host cell amino acids and metabolites. Further, this incorporation was dependent on lysosome activity and observable in bacteria beyond *E. coli*, including pathogens such as *Staphylococcus aureus*. Importantly, the authors observed a noticeable reduction in “recycling” efficiency when living *E. coli* (LEC) were used; rather, MΦ fed LEC adopted a pro-inflammatory phenotype characterized by elevated reactive oxygen species (ROS) production and IL-1β secretion. The authors go on to demonstrate that KEC are enriched in cyclic adenosine monophosphate (cAMP) relative to their living counterparts and that this molecule is readily converted to AMP in host MΦ. This spike in intracellular AMP activates adenosine monophosphate kinase (AMPK), suppressing mechanistic target of rapamycin complex 1 (mTORC1) and committing the host MΦ to a distinct immunometabolic phenotype. Thus, Lesbats et al demonstrate that MΦ discriminate between living and killed bacteria, using this “live or dead zip code” to deliver a more immune-driven or more metabolic-adapted phenotype, respectively (Figure 1).

**Figure 1.**
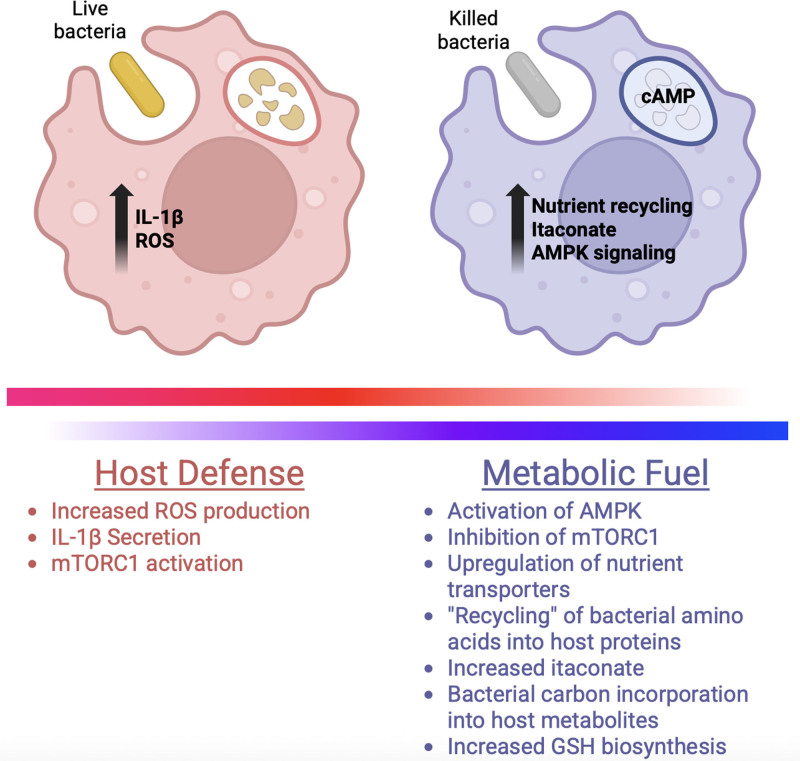
**The fate of live and dead bacteria in phagocytosed macrophages**. Live bacteria (red) trigger a host defense response that results in the activation of mTORC1, increased ROS, and ultimately IL-1β secretion. Macrophage phagocytosis of killed bacteria (blue) elicits a more metabolic recycling phenotype that activates AMPK through elevated cAMP. Such findings suggest that macrophages may “sense” their immune microenvironment and adapt their metabolic state accordingly. AMPK, adenosine monophosphate kinase; cAMP, cyclic adenosine monophosphate; mTORC1, mechanistic target of rapamycin complex 1; ROS, reactive oxygen species.

This work raises a number of interesting questions that the authors, understandably, were not able to fully address in their manuscript. Foremost among these is the uncanny similarity between “recycling” phagocytosis and autophagy, a conserved eukaryotic process of intracellular material degradation ^[6]^. The function of the lysosome as a degradation hub is not unique to phagocytosis; indeed, phagocytic and nonphagocytic cells alike utilize the lysosome for degradation of misfolded proteins, damaged organelles, and so forth during autophagy ^[7]^. The importance of the lysosome in these pathways as a central metabolic regulator in the cell is, in part, through its association with mTORC1 and Rag/Rheb GTP-binding proteins ^[8]^. Autophagy can also involve the targeting of intracellular microbes in a process termed “xenophagy”, a function that bears a striking resemblance to phagocytosis ^[9,10]^. The parallels between autophagy and the phagocytosis-mediated recycling process presented by Lesbats et al can thus be summarized in three ways. The first is the mechanistic overlap between the processes: during certain phagocytic events, autophagy proteins such as MAP1LC3 (LC3), ATG5, ATG12, and BECN1 are recruited to the phagosome to assist with lysosomal fusion in a process termed “LC3-associated phagocytosis” (LAP) ^[9]^. These events are primarily seen in professional phagocytes and in a TLR/Fc receptor-dependent manner. Although LAP was not directly assessed by Lesbats et al, it could be surmised that LAP acts as an extracellular homolog of the intracellular xenophagy in phagocytic-competent cells such as MΦ. Second, both processes return nutrients from degraded bacteria to the host cell. Although autophagy has long been theorized to return nutrients to the cell from degraded cargo, it was only within the last 20 years that this process was demonstrated at a molecular level ^[11,12]^. It is highly probable that microbes degraded through autophagic processes are recycled by the cell, similar to phagocytosis. Finally, the intracellular signaling mechanisms that stimulate the “recycling” phagocytosis phenotype in MΦ are also classic activators of autophagy. AMPK, as an intracellular nutrient sensor, has been shown to potentiate autophagic flux directly through activating phosphorylation of Unc-51 like autophagy activating kinase 1 (ULK1) and inhibitory phosphorylation of the mTORC1 subunit Raptor, among other functions ^[13]^. mTORC1 itself regulates autophagy through inhibitory phosphorylation of ULK1 (and other targets); the activation of AMPK and inhibition of mTORC1 seen in KEC-treated MΦ is a classic signature of starved cells with increased autophagic flux ^[14]^. Thus, “recycling” phagocytosis and autophagy could be considered the molecular equivalent of “kindred spirits”.

Other questions arise from this work, aside from those relating to autophagy. Lesbats et al show that, in addition to MΦ, labeled KEC were efficiently phagocytosed by Ly6G^+^ neutrophils in vivo. This raises the question of whether their observations are broadly applicable to all phagocytic cells. If so, does this alter their innate cell function? Neutrophils, for example, are known to be highly reliant on the pentose phosphate pathway for the generation of NADP(H) and, in turn, the generation of ROS ^[15]^. Perhaps the increased supply of metabolic intermediates provided by killed bacteria might enhance their respiratory burst? Might the phagocytosis of killed versus live bacteria alter neutrophil fate, such as the proclivity to undergo NETosis, through regulation of neutrophil metabolism ^[16]^? Similar questions might be posed for dendritic cells. It has long been understood that the immune system can differentiate between living and dead microbes ^[17]^; however, the precise mechanisms of this discrimination are not fully understood. Dendritic cells in particular are known to present antigen differently depending on whether live or inactivated bacteria are encountered, altering downstream immune responses ^[18]^. Perhaps a form of “metabolic imprinting” exists in these cells as well, and, if so, might it be leveraged for therapeutic use, such as to improve the efficacy of vaccines? It will be exciting to pursue these and other such questions in future studies. Further, the precise role of AMPK in shaping the immunometabolic phenotype of macrophages has yet to be fully understood, as demonstrated by this work. Previous studies demonstrate that AMPK activation restricts protein translation, yet Lesbats et al observe a distinct translational phenotype (glycolysis, innate immunity, etc) in macrophages following phagocytosis of KEC ^[19]^. It is possible that AMPK is responsible for directing particular translational programs; in the present case, AMPK may curtail translational activity that is unrelated to the task at hand of clearing extracellular bacteria.

In summary, this publication offers unique insight into the function of MΦ by demonstrating that phagocytosed bacteria are directly recycled into host cell proteins and metabolites. Further, by showing that this recycling phenotype is dependent on the viability of the phagocytosed bacteria, Lesbats et al reveal that MΦ may “sense” their immune microenvironment and adapt their metabolic state accordingly, either as a soldier “pro-inflammatory” program in response to living bacteria or a more janitorial “clean-up” role when dead microbes are the cargo (Figure 1).

## Conflicts of interest

The authors declare that they have no conflicts of interest.

## Funding

This manuscript was supported by NIH grants DK1047893, DK50189, DK095491, DK103639, and VA Merit BX002182.
